# Supplementary material for: Integrating genomic information and productivity and climate-adaptability traits into a regional white spruce breeding program
Source: PLoS One. 2022 Mar 17;17(3):e0264549. doi: 10.1371/journal.pone.0264549 (PMC8929621; doi:10.1371/journal.pone.0264549)

**S1 Fig. Annual variation in average basal area increment (BAI) of the open-pollinated white spruce families for the period 1995–2016 at each of the three test sites.** The red dashed line represents the year of the drought event and the green shadowed area represents the pre-drought period considered to calculate the Resistance index.


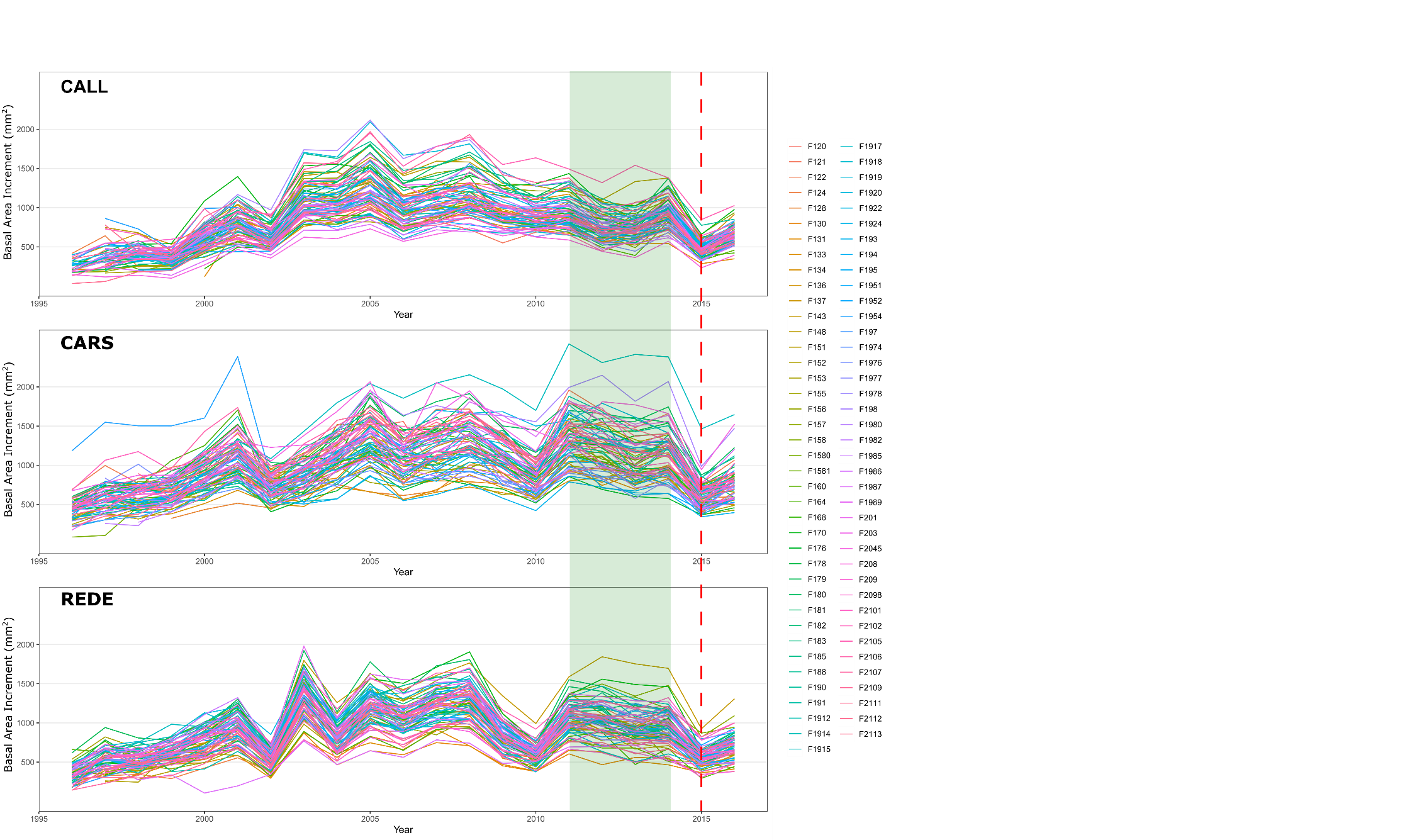

Supplement: S1 Fig — The red dashed line represents the year of the drought event and the green shadowed area represents the pre-drought period considered to calculate the Resistance index. (DOCX) [file pone.0264549.s001.docx]
