# Supplementary material for: Integrating genomic information and productivity and climate-adaptability traits into a regional white spruce breeding program
Source: PLoS One. 2022 Mar 17;17(3):e0264549. doi: 10.1371/journal.pone.0264549 (PMC8929621; doi:10.1371/journal.pone.0264549)

**S2 Fig. Density distribution for the studied traits in white spruce in each of the three test sites.** Logarithmic transformations were applied to MFA and all monoterpene compounds to improve data normality. Abbreviations used for the traits and sites are described, respectively, in the text and Table 1.


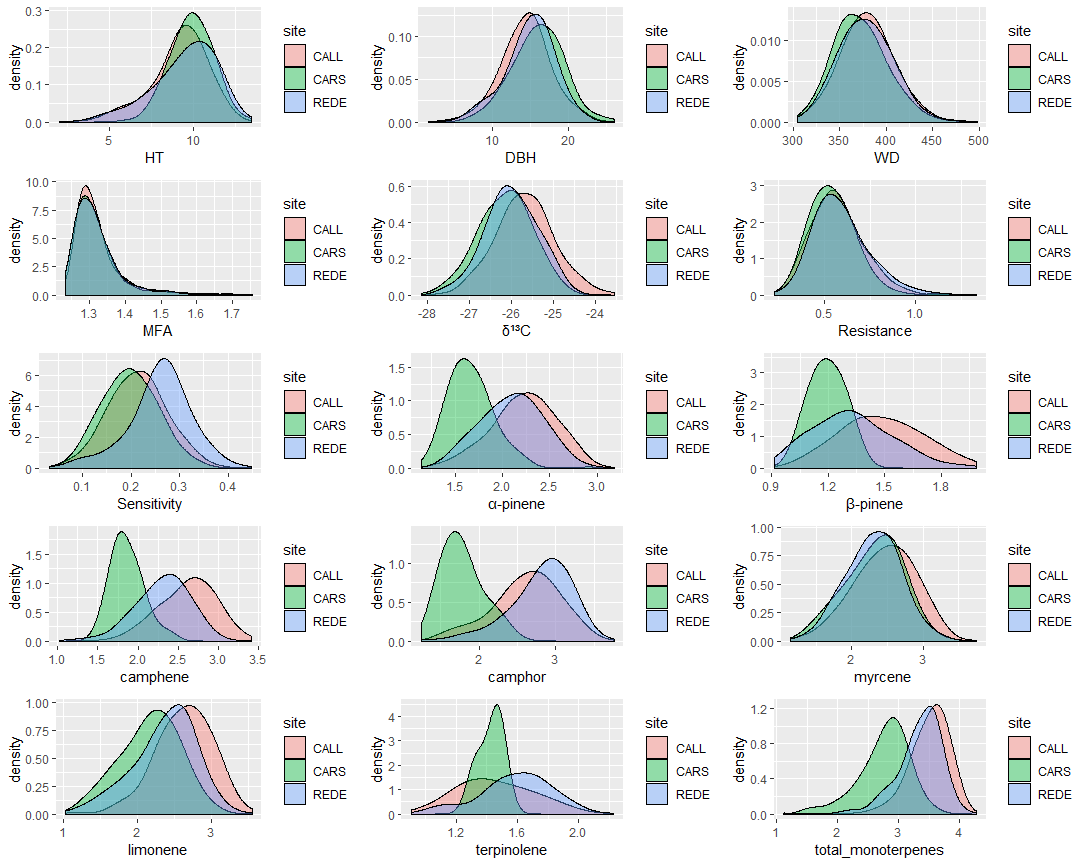

Supplement: S2 Fig — Logarithmic transformations were applied to MFA and all monoterpene compounds to improve data normality. Abbreviations used for the traits and sites are described, respectively, in the text and Table 1. (DOCX) [file pone.0264549.s002.docx]
