# Supplementary material for: Integrating genomic information and productivity and climate-adaptability traits into a regional white spruce breeding program
Source: PLoS One. 2022 Mar 17;17(3):e0264549. doi: 10.1371/journal.pone.0264549 (PMC8929621; doi:10.1371/journal.pone.0264549)
Supplement: S3 Fig — Distribution of the number of pairwise additive relationships (excluding the diagonal elements) from the pedigree (after pedigree correction, left) and genomic (right) relationship matrices. Note that y-axis (Frequency) were cut at 40,000 (A-matrix, out of 2,343,490) and at 10,000 (G-matrix, out of 1,555,212) in order to more clearly visualize the differences between relationship matrices. (DOCX) [file pone.0264549.s003.docx]

**S3 Fig.** **Pedigree and genomic relationships.** Distribution of the number of pairwise additive relationships (excluding the diagonal elements) from the pedigree (after pedigree correction, left) and genomic (right) relationship matrices for the white spruce population studied. Note that *y*-axis (Frequency) were cut at 40,000 (***A***-matrix, out of 2,343,490) and at 10,000 (***G***-matrix, out of 1,555,212) in order to more clearly visualize the differences between relationship matrices.

| ***A***-matrix | ***G***-matrix |
| --- | --- |
| 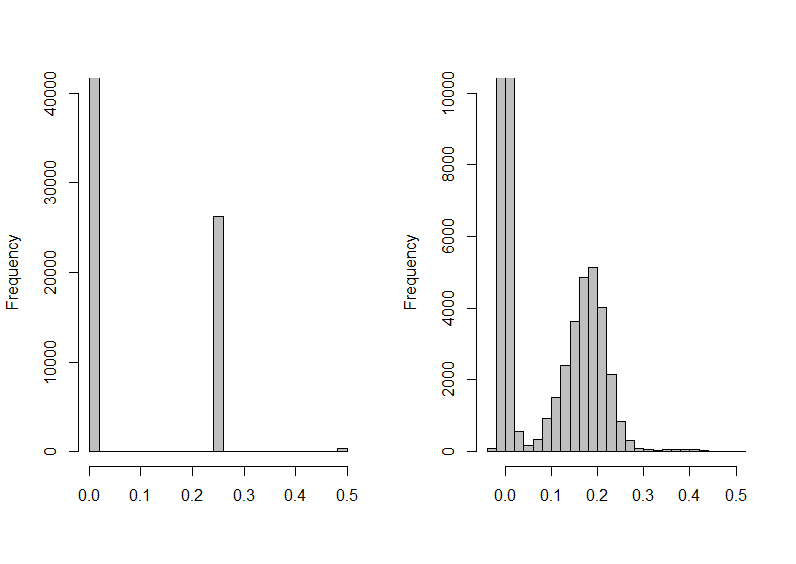 | |
