# Supplementary material for: Integrating genomic information and productivity and climate-adaptability traits into a regional white spruce breeding program
Source: PLoS One. 2022 Mar 17;17(3):e0264549. doi: 10.1371/journal.pone.0264549 (PMC8929621; doi:10.1371/journal.pone.0264549)
Supplement: S4 Fig — Abbreviations used for the sites are described in the Table 1. (DOCX) [file pone.0264549.s004.docx]

**S4 Fig. Scatter plot between estimated genetic correlation between pairs of traits from the pedigree- (*A*-matrix) and genomic-based (*G*-matrix) relationship matrices in each of the three white spruce test sites.** Abbreviations used for the sites are described in the Table 1.

| **** |
| --- |
