# Supplementary material for: Integrating genomic information and productivity and climate-adaptability traits into a regional white spruce breeding program
Source: PLoS One. 2022 Mar 17;17(3):e0264549. doi: 10.1371/journal.pone.0264549 (PMC8929621; doi:10.1371/journal.pone.0264549)
Supplement: S1 Table — Abbreviations used for the traits and sites are described, respectively, in the text and Table 1. (DOCX) [file pone.0264549.s006.docx]

**S1 Table. Estimated genetic correlations (and approximate standard errors) between the different traits from the multiple-trait analysis using the pedigree- (*A*-matrix, above diagonal) and genomic-based (*G*-matrix, below diagonal) relationship matrices for white spruce in each of the three test sites.** Abbreviations used for the traits and sites are described, respectively, in the text and Table 1.

| **CALL** | | | | | | | | | | | | | | | |
| --- | --- | --- | --- | --- | --- | --- | --- | --- | --- | --- | --- | --- | --- | --- | --- |
|  | **HT** | **DBH** | **WD** | **MFA** | **Resistance** | **Sensitivity** | **δ^13^C** | **α-pinene** | **β-pinene** | **pamphene** | **camphor** | **myrcene** | **limonene** | **terpinolene** | **total monoterpenes** |
| **HT** |  | 0.87 (0.01) | 0.06 (0.04) | -0.22 (0.03) | -0.48 (0.03) | 0.64 (0.02) | 0.29 (0.03) | 0.19 (0.03) | 0.14 (0.04) | 0.15 (0.04) | 0.21 (0.03) | 0.22 (0.03) | 0.27 (0.03) | 0.23 (0.03) | 0.27 (0.03) |
| **DBH** | 0.89 (0.01) |  | -0.12 (0.04) | 0.15 (0.04) | -0.32 (0.03) | 0.60 (0.02) | 0.35 (0.03) | 0.12 (0.04) | 0.12 (0.04) | 0.11 (0.04) | 0.26 (0.03) | 0.28 (0.03) | 0.18 (0.04) | 0.21 (0.03) | 0.25 (0.03) |
| **WD** | 0.06 (0.04) | -0.16 (0.04) |  | -0.19 (0.03) | -0.26 (0.03) | -0.15 (0.04) | 0.20 (0.03) | 0.18 (0.03) | 0.19 (0.04) | 0.16 (0.04) | 0.12 (0.04) | 0.22 (0.03) | 0.15 (0.04) | 0.24 (0.03) | 0.19 (0.03) |
| **MFA*^b^*** | -0.23 (0.04) | 0.11 (0.04) | -0.10 (0.04) |  | 0.39 (0.03) | 0.03 (0.04) | -0.06 (0.04) | 0.04 (0.04) | 0.10 (0.04) | 0.07 (0.04) | 0.37 (0.03) | 0.09 (0.04) | -0.01 (0.04) | 0.12 (0.04) | 0.08 (0.04) |
| **Resistance** | -0.40 (0.03) | -0.18 (0.04) | -0.17 (0.04) | 0.51 (0.03) |  | -0.20 (0.03) | -0.11 (0.04) | -0.32 (0.03) | -0.27 (0.03) | -0.27 (0.03) | -0.24 (0.03) | -0.06 (0.04) | -0.13 (0.04) | -0.31 (0.03) | -0.25 (0.03) |
| **Sensitivity** | 0.50 (0.03) | 0.40 (0.03) | -0.05 (0.04) | -0.21 (0.04) | -0.09 (0.04) |  | -0.01 (0.04) | 0.51 (0.03) | 0.51 (0.03) | 0.53 (0.03) | 0.60 (0.02) | 0.36 (0.03) | 0.49 (0.03) | 0.50 (0.03) | 0.63 (0.02) |
| **δ^13^C** | 0.26 (0.04) | 0.29 (0.04) | 0.25 (0.04) | -0.09 (0.04) | -0.01 (0.04) | 0.12 (0.04) |  | 0.07 (0.04) | 0.09 (0.04) | 0.05 (0.04) | 0.19 (0.03) | 0.37 (0.03) | 0.07 (0.04) | 0.20 (0.03) | 0.17 (0.03) |
| **α-pinene*^b^*** | 0.00 (0.04) | -0.11 (0.04) | 0.20 (0.04) | -0.07 (0.04) | -0.36 (0.04) | 0.42 (0.03) | 0.03 (0.04) |  | 0.94 (0) | 0.83 (0.01) | 0.67 (0.02) | 0.64 (0.02) | 0.90 (0.01) | 0.96 (0.00) | 0.94 (0.00) |
| **β-pinene*^b^*** | -0.02 (0.04) | -0.09 (0.04) | 0.20 (0.04) | -0.01 (0.04) | -0.29 (0.04) | 0.40 (0.03) | 0.03 (0.04) | 0.95 (0.00) |  | 0.93 (0.01) | 0.64 (0.02) | 0.57 (0.02) | 0.84 (0.01) | 0.88 (0.01) | 0.93 (0.01) |
| **camphene*^b^*** | -0.03 (0.04) | -0.11 (0.04) | 0.16 (0.04) | -0.04 (0.04) | -0.32 (0.04) | 0.43 (0.03) | 0.00 (0.04) | 0.86 (0.01) | 0.94 (0.00) |  | 0.63 (0.02) | 0.45 (0.03) | 0.75 (0.02) | 0.77 (0.01) | 0.88 (0.01) |
| **camphor*^b^*** | 0.04 (0.04) | 0.00 (0.04) | 0.16 (0.04) | 0.19 (0.04) | -0.29 (0.04) | 0.44 (0.03) | 0.06 (0.04) | 0.77 (0.02) | 0.74 (0.02) | 0.73 (0.02) |  | 0.65 (0.02) | 0.59 (0.03) | 0.79 (0.02) | 0.70 (0.02) |
| **myrcene*^b^*** | 0.09 (0.04) | 0.04 (0.04) | 0.20 (0.04) | -0.07 (0.04) | -0.10 (0.04) | 0.33 (0.04) | 0.25 (0.04) | 0.72 (0.02) | 0.66 (0.02) | 0.55 (0.03) | 0.54 (0.03) |  | 0.65 (0.02) | 0.74 (0.02) | 0.73 (0.02) |
| **limonene*^b^*** | 0.12 (0.04) | -0.02 (0.04) | 0.21 (0.04) | -0.16 (0.04) | -0.18 (0.04) | 0.48 (0.03) | 0.02 (0.04) | 0.89 (0.01) | 0.84 (0.01) | 0.75 (0.02) | 0.49 (0.03) | 0.73 (0.02) |  | 0.86 (0.01) | 0.88 (0.01) |
| **terpinolene*^b^*** | 0.01 (0.04) | -0.05 (0.04) | 0.23 (0.04) | 0.08 (0.04) | -0.32 (0.04) | 0.36 (0.03) | 0.07 (0.04) | 0.96 (0.00) | 0.90 (0.01) | 0.81 (0.01) | 0.70 (0.02) | 0.76 (0.02) | 0.82 (0.01) |  | 0.96 (0.00) |
| **total monoterpenes*^b^*** | 0.09 (0.04) | 0.01 (0.04) | 0.19 (0.04) | -0.05 (0.04) | -0.30 (0.04) | 0.54 (0.03) | 0.10 (0.04) | 0.96 (0.00) | 0.94 (0.00) | 0.90 (0.01) | 0.78 (0.02) | 0.78 (0.02) | 0.87 (0.01) | 0.95 (0.00) |  |
| **CARS** | | | | | | | | | | | | | | | |
| **HT** |  | 0.93 (0.01) | -0.35 (0.04) | 0.23 (0.05) | -0.28 (0.04) | -0.31 (0.04) | 0.62 (0.03) | -0.23 (0.05) | *a* | -0.20 (0.05) | -0.43 (0.04) | -0.28 (0.04) | -0.23 (0.05) | *a* | -0.24 (0.05) |
| **DBH** | 0.89 (0.01) |  | -0.40 (0.04) | 0.33 (0.04) | -0.22 (0.05) | -0.25 (0.05) | 0.70 (0.03) | -0.30 (0.04) | *a* | -0.31 (0.04) | -0.45 (0.04) | -0.43 (0.04) | -0.41 (0.04) | *a* | -0.41 (0.05) |
| **WD** | -0.40 (0.05) | -0.25 (0.05) |  | -0.42 (0.04) | -0.15 (0.05) | -0.12 (0.05) | 0.06 (0.05) | 0.41 (0.04) | *a* | 0.59 (0.03) | 0.09 (0.05) | 0.69 (0.03) | 0.61 (0.03) | *a* | 0.65 (0.04) |
| **MFA*^b^*** | 0.16 (0.06) | 0.33 (0.05) | -0.11 (0.06) |  | 0.77 (0.02) | -0.04 (0.05) | 0.09 (0.05) | 0.44 (0.04) | *a* | 0.02 (0.05) | 0.34 (0.04) | -0.08 (0.05) | 0.11 (0.05) | *a* | 0.09 (0.05) |
| **Resistance** | -0.37 (0.05) | -0.27 (0.05) | -0.08 (0.06) | 0.76 (0.02) |  | 0.00 (0.05) | -0.22 (0.05) | 0.52 (0.04) | *a* | 0.28 (0.05) | 0.56 (0.03) | 0.26 (0.05) | 0.29 (0.04) | *a* | 0.37 (0.05) |
| **Sensitivity** | -0.08 (0.06) | -0.1 (0.06) | 0.15 (0.06) | 0.19 (0.05) | 0.38 (0.05) |  | 0.19 (0.05) | 0.01 (0.05) | *a* | -0.02 (0.05) | -0.33 (0.04) | -0.1 (0.05) | 0.05 (0.05) | *a* | -0.05 (0.05) |
| **δ^13^C** | 0.55 (0.04) | 0.66 (0.03) | 0.15 (0.06) | 0.10 (0.06) | -0.34 (0.04) | 0.24 (0.05) |  | -0.08 (0.05) | *a* | -0.06 (0.05) | -0.69 (0.03) | -0.17 (0.05) | -0.09 (0.05) | *a* | -0.15 (0.05) |
| **α-pinene*^b^*** | -0.37 (0.05) | -0.30 (0.05) | 0.55 (0.04) | 0.56 (0.04) | 0.64 (0.03) | 0.4 (0.05) | -0.04 (0.06) |  | *a* | 0.72 (0.02) | 0.64 (0.03) | 0.45 (0.04) | 0.89 (0.01) | *a* | 0.80 (0.04) |
| **β-pinene*^b^*** | *a* | *a* | *a* | *a* | *a* | *a* | *a* | *a* |  | *a* | *a* | *a* | *a* | *a* | *a* |
| **camphene*^b^*** | -0.34 (0.05) | -0.27 (0.05) | 0.65 (0.03) | 0.39 (0.05) | 0.49 (0.04) | 0.61 (0.04) | -0.06 (0.06) | 0.78 (0.02) | *a* |  | 0.39 (0.04) | 0.5 (0.04) | 0.77 (0.02) | *a* | 0.87 (0.04) |
| **camphor*^b^*** | -0.49 (0.04) | -0.48 (0.04) | 0.02 (0.06) | 0.22 (0.05) | 0.39 (0.05) | 0.09 (0.06) | -0.65 (0.03) | 0.49 (0.04) | *a* | 0.24 (0.05) |  | 0.02 (0.06) | 0.44 (0.05) | *a* | 0.43 (0.05) |
| **myrcene*^b^*** | -0.31 (0.05) | -0.36 (0.05) | 0.58 (0.04) | 0.08 (0.06) | 0.28 (0.05) | 0.39 (0.05) | -0.12 (0.06) | 0.57 (0.04) | *a* | 0.61 (0.04) | 0.16 (0.05) |  | 0.7 (0.02) | *a* | 0.81 (0.04) |
| **limonene*^b^*** | -0.31 (0.05) | -0.37 (0.05) | 0.62 (0.03) | 0.17 (0.06) | 0.32 (0.05) | 0.58 (0.04) | -0.02 (0.06) | 0.86 (0.02) | *a* | 0.75 (0.03) | 0.47 (0.04) | 0.68 (0.03) |  | *a* | 0.93 (0.04) |
| **terpinolene*^b^*** | *a* | *a* | *a* | *a* | *a* | *a* | *a* | *a* | *a* | *a* | *a* | *a* | *a* |  | *a* |
| **total monoterpenes*^b^*** | -0.33 (0.05) | -0.36 (0.05) | 0.65 (0.05) | 0.32 (0.06) | 0.46 (0.05) | 0.56 (0.05) | -0.11 (0.06) | 0.85 (0.05) | *a* | 0.91 (0.04) | 0.27 (0.06) | 0.84 (0.05) | 0.89 (0.04) | *a* |  |
| **REDE** | | | | | | | | | | | | | | | |
| **HT** |  | 0.93 (0.01) | -0.11 (0.04) | -0.47 (0.03) | -0.65 (0.02) | 0.68 (0.02) | 0.40 (0.03) | -0.15 (0.04) | -0.18 (0.04) | -0.18 (0.04) | -0.21 (0.03) | 0.08 (0.04) | -0.12 (0.04) | -0.17 (0.04) | -0.14 (0.04) |
| **DBH** | 0.91 (0.01) |  | -0.26 (0.03) | -0.31 (0.03) | -0.61 (0.02) | 0.78 (0.01) | 0.21 (0.03) | -0.16 (0.04) | -0.20 (0.03) | -0.20 (0.03) | -0.27 (0.03) | 0.08 (0.04) | -0.1 (0.04) | -0.24 (0.03) | -0.16 (0.04) |
| **WD** | -0.12 (0.04) | -0.28 (0.04) |  | -0.34 (0.03) | -0.15 (0.04) | -0.04 (0.04) | 0.32 (0.03) | 0.09 (0.04) | 0.15 (0.04) | 0.08 (0.04) | 0.19 (0.04) | -0.05 (0.04) | 0.01 (0.04) | 0.13 (0.04) | 0.11 (0.04) |
| **MFA*^b^*** | -0.50 (0.03) | -0.30 (0.04) | -0.31 (0.04) |  | 0.55 (0.03) | -0.41 (0.03) | -0.33 (0.03) | -0.16 (0.04) | -0.13 (0.04) | -0.16 (0.04) | -0.15 (0.04) | 0.10 (0.04) | -0.27 (0.03) | -0.2 (0.04) | -0.21 (0.04) |
| **Resistance** | -0.64 (0.02) | -0.55 (0.03) | -0.15 (0.04) | 0.64 (0.02) |  | -0.81 (0.01) | -0.37 (0.04) | 0.10 (0.04) | 0.06 (0.04) | 0.10 (0.04) | 0.08 (0.04) | 0.06 (0.04) | 0.05 (0.04) | 0.09 (0.04) | 0.04 (0.04) |
| **Sensitivity** | 0.64 (0.02) | 0.77 (0.02) | -0.01 (0.04) | -0.45 (0.03) | -0.79 (0.02) |  | 0.21 (0.04) | -0.04 (0.04) | -0.06 (0.04) | -0.06 (0.04) | -0.04 (0.04) | 0.07 (0.04) | 0.04 (0.04) | -0.12 (0.04) | 0.03 (0.04) |
| **δ^13^C** | 0.40 (0.03) | 0.20 (0.04) | 0.27 (0.04) | -0.41 (0.03) | -0.32 (0.03) | 0.20 (0.04) |  | 0.01 (0.04) | -0.01 (0.04) | 0.00 (0.04) | 0.07 (0.04) | 0.26 (0.03) | 0.10 (0.04) | -0.03 (0.04) | 0.09 (0.04) |
| **α-pinene*^b^*** | -0.21 (0.04) | -0.17 (0.04) | -0.06 (0.04) | -0.11 (0.04) | 0.13 (0.04) | -0.06 (0.04) | -0.02 (0.04) |  | 0.98 (0.00) | 0.99 (0.00) | 0.87 (0.01) | 0.81 (0.01) | 0.91 (0.01) | 0.95 (0.00) | 0.97 (0.00) |
| **β-pinene*^b^*** | -0.23 (0.04) | -0.20 (0.04) | -0.02 (0.04) | -0.08 (0.04) | 0.08 (0.04) | -0.06 (0.04) | -0.03 (0.04) | 0.98 (0.00) |  | 0.98 (0.00) | 0.87 (0.01) | 0.80 (0.01) | 0.88 (0.01) | 0.95 (0.00) | 0.95 (0.00) |
| **camphene*^b^*** | -0.22 (0.04) | -0.20 (0.04) | -0.07 (0.04) | -0.11 (0.04) | 0.13 (0.04) | -0.09 (0.04) | -0.01 (0.04) | 1.00 (0.00) | 0.98 (0.00) |  | 0.90 (0.01) | 0.81 (0.01) | 0.90 (0.01) | 0.96 (0.00) | 0.98 (0.00) |
| **camphor*^b^*** | -0.28 (0.04) | -0.30 (0.04) | 0.01 (0.04) | -0.06 (0.04) | 0.09 (0.04) | -0.04 (0.04) | 0.00 (0.04) | 0.84 (0.01) | 0.84 (0.01) | 0.86 (0.01) |  | 0.75 (0.02) | 0.80 (0.01) | 0.87 (0.01) | 0.94 (0.00) |
| **myrcene*^b^*** | 0.00 (0.04) | 0.08 (0.04) | -0.16 (0.04) | 0.13 (0.04) | 0.09 (0.04) | 0.09 (0.04) | 0.23 (0.04) | 0.81 (0.01) | 0.81 (0.01) | 0.81 (0.01) | 0.76 (0.02) |  | 0.71 (0.02) | 0.75 (0.02) | 0.82 (0.01) |
| **limonene*^b^*** | -0.17 (0.04) | -0.12 (0.04) | -0.10 (0.04) | -0.25 (0.04) | 0.04 (0.04) | 0.04 (0.04) | 0.06 (0.04) | 0.91 (0.01) | 0.88 (0.01) | 0.89 (0.01) | 0.83 (0.01) | 0.72 (0.02) |  | 0.84 (0.01) | 0.92 (0.01) |
| **terpinolene*^b^*** | -0.18 (0.04) | -0.23 (0.04) | -0.01 (0.04) | -0.17 (0.04) | 0.08 (0.04) | -0.11 (0.04) | -0.02 (0.04) | 0.94 (0.00) | 0.95 (0.00) | 0.96 (0.00) | 0.91 (0.01) | 0.73 (0.02) | 0.82 (0.01) |  | 0.95 (0.00) |
| **total monoterpenes*^b^*** | -0.21 (0.04) | -0.17 (0.04) | -0.07 (0.04) | -0.14 (0.04) | 0.07 (0.04) | 0.02 (0.04) | 0.06 (0.04) | 0.97 (0.00) | 0.95 (0.00) | 0.98 (0.00) | 0.92 (0.01) | 0.83 (0.01) | 0.91 (0.01) | 0.94 (0.01) |  |

**NOTE:*^a^*** Correlations and their approximate standard errors were not estimated at the CARS site due to insufficient phenotypic data.

***^b^*** Transformed data used for estimates.
