# Supplementary material for: Integrating genomic information and productivity and climate-adaptability traits into a regional white spruce breeding program
Source: PLoS One. 2022 Mar 17;17(3):e0264549. doi: 10.1371/journal.pone.0264549 (PMC8929621; doi:10.1371/journal.pone.0264549)
Supplement: S2 Table — Abbreviations used for the traits and sites are described, respectively, in the text and Table 1. (DOCX) [file pone.0264549.s007.docx]

**S2 Table. Estimated genetic correlations (and approximate standard errors) between the different sites from the multiple-site analysis using the pedigree- (A-matrix, above diagonal) and genomic-based (G-matrix, below diagonal) relationship matrices for white spruce in each of the three test sites.** Abbreviations used for the traits and sites are described, respectively, in the text and Table 1.

| **trait** | **Site** | **CALL** | **CARS** | **REDE** |
| --- | --- | --- | --- | --- |
| **HT** | **CALL** |  | 0.14 (0.24) | 0.87 (0.12) |
|  | **CARS** | 0.26 (0.31) |  | 0.28 (0.23) |
|  | **REDE** | 0.79 (0.19) | 0.28 (0.28) |  |
| **DBH** | **CALL** |  | 0.24 (0.16) | 0.90 (0.03) |
|  | **CARS** | 0.19 (1.00) |  | 0.51 (0.13) |
|  | **REDE** | 0.86 (1.57) | 0.46 (0.78) |  |
| **WD** | **CALL** |  | 0.97 (0.02) | 0.98 (0.01) |
|  | **CARS** | 0.97 (0.03) |  | 0.98 (0.02) |
|  | **REDE** | 0.98 (0.02) | 0.98 (0.02) |  |
| **MFA*^c^*** | **CALL** |  | 0.42 (0.30) | 0.92 (0.07) |
|  | **CARS** | 0.37 (0.42) |  | 0.66 (0.25) |
|  | **REDE** | 0.89 (0.11) | 0.67 (0.35) |  |
| **Resistence** | **CALL** |  | -0.02 (0.98) | 0.67 (0.23) |
|  | **CARS** | 0.02 (0.57) |  | 0.69 (0.38) |
|  | **REDE** | 0.68 (0.24) | 0.70 (0.38) |  |
| **Sensitivity** | **CALL** |  | 0.46 (0.25) | 0.93 (0.03) |
|  | **CARS** | 0.22 (0.40) |  | 0.27 (0.28) |
|  | **REDE** | 0.93 (0.04) | -0.01 (0.42) |  |
| **δ^13^C** | **CALL** |  | 0.95 (0.02) | 0.97 (0.01) |
|  | **CARS** | 0.88 (0.06) |  | 0.93 (0.03) |
|  | **REDE** | 0.97 (0.02) | 0.80 (0.10) |  |
| **α-pinene*^c^*** | **CALL** |  | 0.86 (0.07) | 0.63 (0.13) |
|  | **CARS** | 0.86 (0.07) |  | 0.43 (0.20) |
|  | **REDE** | 0.61 (0.14) | 0.43 (0.21) |  |
| **β-pinene*^c^*** | **CALL** |  | ***b*** | 0.60 (0.79) |
|  | **CARS** | ***b*** |  | ***b*** |
|  | **REDE** | 0.65 (1.41) | ***b*** |  |
| **camphene*^c^*** | **CALL** |  | 0.60 (0.09) | 0.54 (0.10) |
|  | **CARS** | 0.68 (0.09) |  | 0.84 (0.05) |
|  | **REDE** | 0.60 (0.10) | 0.82 (0.06) |  |
| **camphor*^c^*** | **CALL** |  | ***b*** | 0.33 (0.35) |
|  | **CARS** | ***b*** |  | ***B*** |
|  | **REDE** | 0.46 (0.53) | ***b*** |  |
| **myrcene*^c^*** | **CALL** |  | 0.89 (0.03) | 0.91 (0.03) |
|  | **CARS** | 0.85 (0.47) |  | 0.81 (0.05) |
|  | **REDE** | 0.84 (0.39) | 0.74 (0.67) |  |
| **limonene*^c^*** | **CALL** |  | 0.91 (0.04) | 0.94 (0.03) |
|  | **CARS** | 0.93 (0.03) |  | 0.84 (0.06) |
|  | **REDE** | 0.91 (0.04) | 0.88 (0.05) |  |
| **terpinolene*^c^*** | **CALL** |  | ***b*** | 0.15 (0.44) |
|  | **CARS** | ***b*** |  | ***b*** |
|  | **REDE** | 0.4 (0.73) | ***b*** |  |
| **total** | **CALL** |  | 0.87 (0.06) | 0.96 (0.02) |
| **monoterpene*^c^*** | **CARS** | 0.87 (0.07) |  | 0.87 (0.06) |
|  | **REDE** | 0.95 (0.02) | 0.86 (0.07) |  |

**NOTE: *^a^*** Correlation and their approximate standard errors were not estimated due to convergence problems

***^b^*** Correlations and their approximate standard errors were not estimated at the CARS site due to insufficient phenotypic data.

***^c^*** Transformed data used for estimates.
