# Supplementary material for: Integrating genomic information and productivity and climate-adaptability traits into a regional white spruce breeding program
Source: PLoS One. 2022 Mar 17;17(3):e0264549. doi: 10.1371/journal.pone.0264549 (PMC8929621; doi:10.1371/journal.pone.0264549)
Supplement: S1 Text — (DOCX) [file pone.0264549.s008.docx]

**S1 Text. Chemical analysis**

Frozen and ground tissue was extracted twice with 0.5 ml of hexane with 0.004% pentadecane (internal standard), vortexing for 30 sec, sonicated for 10 min, and centrifuging for 15 min. We used a Gas Chromatograph/Mass Spectrometer (GC/MS, Agilent 7890A/5062C, Agilent Tech., Santa Clara, CA, USA) using an HP-Innowax column for identification of chemicals from a subset of samples. To quantify chemicals for all samples, we used Gas Chromatograph/Flame Ionization Detector (GC/FID, Agilent 7890B). The analytical method used for both GC/MS and FID was the following: sample extract (1 μl) was injected with a split injection (10:1) into the GC equipped with a HP-Innowax column (I.D. 0.25 mm, length 30 m) (Agilent Techn.) with helium carrier gas flow at 1.1 ml min-1, and a temperature of 40°C for 1 min, increased to 55°C by 30°C min-1 and held for 0.5 min, increased to 122°C by 8°C min-1 and held for 2 min, increased to 200°C by 10°C min-1, and then to 260°C by 20°C min-1 and held for 1 min. The following authentic standards were used for identification and quantification: borneol, α-terpinene, γ-terpinene, α-terpineol (Sigma-Aldrich, St. Louis, MO, USA), 3-carene, terpinolene, α-pinene, β-pinene, limonene, myrcene, camphene, p-cymene, 4-allylanisole, camphor (Fluka, Sigma-Aldrich, Buchs, Switzerland), bornyl acetate (SAFC Supply Solutions, St. Louis, MO, USA), and β-phellandrene (Erbilgin lab).
